# Signature Construction and Molecular Subtype Identification Based on Pyroptosis-Related Genes for Better Prediction of Prognosis in Hepatocellular Carcinoma

**DOI:** 10.1155/2022/4494713

**Published:** 2022-01-11

**Authors:** Ji Chen, Qiqi Tao, Zhichao Lang, Yuxiang Gao, Yan Jin, Xiaoqi Li, Yajing Wang, Yuxiao Zhang, Suhui Yu, Boyu Lv, Zhengping Yu, Changyong Lin

**Affiliations:** ^1^Key Laboratory of Diagnosis and Treatment of Severe Hepato-Pancreatic Diseases of Zhejiang Province, The First Affiliated Hospital of Wenzhou Medical University, Wenzhou 325000, China; ^2^Department of Hepatobiliary Surgery, The First Affiliated Hospital of Wenzhou Medical University, Wenzhou 325000, China; ^3^Department of Pathology, The First Affiliated Hospital of Wenzhou Medical University, Wenzhou 325000, China; ^4^Department of General Surgery, Wenzhou Hospital of Traditional Chinese Medicine Affiliated to Zhejiang Chinese Medical University, Wenzhou 325000, China

## Abstract

Hepatocellular carcinoma (HCC) is one of the most common malignancies worldwide. However, there is a lack of adequate means of treatment prognostication for HCC. Pyroptosis is a newly discovered way of programmed cell death. However, the prognostic role of pyroptosis in HCC has not been thoroughly investigated. Here, we generated a novel prognostic signature to evaluate the prognostic value of pyroptosis-related genes (PRGs) using the data from The Cancer Genome Atlas (TCGA) database. The accuracy of the signature was validated using survival analysis through the International Cancer Genome Consortium cohort (*n* = 231) and the First Affiliated Hospital of Wenzhou Medical University cohort (*n* = 180). Compared with other clinical factors, the risk score of the signature was found to be associated with better patient outcomes. The enrichment analysis identified multiple pathways related with pyroptosis in HCC. Furthermore, drug sensitivity testing identified six potential chemotherapeutic agents to provide possible treatment avenues. Interestingly, patients with low risk were confirmed to be associated with lower tumor mutation burden (TMB). However, patients at high risk were found to have a higher count of immune cells. Consensus clustering was performed to identify two main molecular subtypes (named clusters A and B) based on the signature. It was found that compared with cluster B, better survival outcomes and lower TMB were observed in cluster A. In conclusion, signature construction and molecular subtype identification of PRGs could be used to predict the prognosis of HCC, which may provide a specific reference for the development of novel biomarkers for HCC treatment.

## 1. Introduction

The etiology and molecular mechanism of hepatocellular carcinoma (HCC), a significant subtype of liver cancer, remain largely unknown [1]. HCC ranks fourth among the most lethal cancers and lacks appropriate treatment [2]. In the United States, the 5-year survival rate for patients with HCC is approximately 18% [3]. In addition, HCC is a highly heterogeneous disease, which has been documented at interpatient, intertumoral, and intertumoral level [4–6]. Previous studies have indicated that hepatocyte death chronically promotes HCC, but the related molecular mechanism is not well defined [7]. Thus, both the poor prognostic conditions and the unknown molecular mechanisms indicate the urgent need to improve the prognosis of HCC.

Pyroptosis, a type of programmed cell death, manifests as the continuous swelling of cells until the cell membrane ruptures, resulting in the release of intracellular contents, followed by the activation of a strong inflammatory response [8]. As programmed necrosis mediated by gasdermin, pyroptosis is different from other cell death modalities, such as apoptosis and necrosis in the morphological features, occurrence, and regulatory mechanism [9]. Pyroptosis has been reported to take a part in tumor genesis, invasion, and metastasis [10]. Some studies have been found that pyroptosis is widely involved in the occurrence and development of various types of diseases [11, 12] and could inhibit the onset of associated diseases to improve the overall survival (OS) of patients [13]. Pyroptosis has also been confirmed to have strong associations with multiple known biomarkers [14, 15]. A recent study demonstrated the critical regulatory role of pyroptosis in the tumor microenvironment (TME), which provides new therapeutic insights for cancer treatment [16]. Therefore, an in-depth study of pyroptosis may help understand its role in the occurrence and development of cancers including HCC and provide new ideas for the clinical prevention and treatment [17, 18].

The tumor mutation burden (TMB) is the total number of substitution and insertion/deletion mutations that occur/megabase in the exon coding regions of the genes evaluated in one tumor sample. TMB, as a biomarker for high-frequency mutations and neoantigens, plays an important role in the immunotherapy in various cancers [19, 20]. Elevated TMB in tumor cells have more neoantigens, resulting in an increase in antitumor T cells in the TME. Interestingly, patients with high TMB have a higher probability in the response to tumor immunotherapy [21]. Dysregulated TMB has also been reported to be involved in the prognosis of cancers [22]. Different cancer species vary in the expression of TMB [23]. In HCC, the higher of TMB means the worse in the OS of patients [24]. However, whether TMB could serve as a biomarker in HCC is still unclear.

Herein, we constructed a novel prognostic gene signature to explore the prognostic value of pyroptosis-related genes (PRGs) and the relationships with tumor mutation and immunotherapy. Our data suggested that risk score was identified as an independent prognostic factor. Notably, the prognostic prediction of our risk signature was also confirmed by the International Cancer Genome Consortium (ICGC) cohort (*n* = 231) and the First Affiliated Hospital of Wenzhou Medical University (FAHWMU) cohort (*n* = 180). Finally, the effects of risk scores and molecular subtypes on TMB and immune filtration were explored to further evaluate the value of the signature in molecular therapy.

## 2. Materials and Methods

### 2.1. Data Preparation

The RNA sequencing data and relevant clinical characteristics of 371 HCC patients were downloaded from TCGA database (https://portal.gdc.cancer.gov/). We matched the RNA sequencing data and clinical features according to each patient's ID number and excluded six follow-up tumor samples. Thus, TCGA cohort with 365 HCC patients was finally enrolled as the training cohort. The other 231 patients with HCC, along with their RNA-seq data and clinical features, were obtained from the ICGC database (https://dcc.icgc.org/projects/LIRI-JP/). HCC patients obtained from the ICGC database were derived from a subset of Japanese population with HBV or HCV infection [25]. Thus, the ICGC cohort was used as the testing cohort. The data of TCGA cohort and ICGC cohort were downloaded from public databases; thus, our study followed the public data access policies; there was no ethical relationship involved. The FAHWMU cohort (*n* = 180) was obtained from the First Affiliated Hospital of Wenzhou Medical University (Wenzhou, China). HCC samples in the FAHWMU cohort were collected from 2010 to 2020, and OS time was used as the main indicator of the survival time. The collection of this cohort was reviewed and approved by the human research ethics committee of the First Affiliated Hospital of Wenzhou Medical University. The patients/participants provided their written informed consent to participate in this study. All the clinical characters of these patients with HCC are shown in Table 1. Meanwhile, 55 pyroptosis-related genes (PRGs) used in this study were extracted from the MsigDB database (http://www.gsea-msigdb.org/gsea/msigdb/) and prior reviews (Table S1) [26–28]. In addition, the data of TMB and immune infiltration of HCC patients in TCGA cohort were also obtained from TCGA database.

### 2.2. Identification of Prognosis-Related DEPRGs

The Wilcoxon rank sum test was used for the differential analysis to identify differentially expressed pyroptosis-related genes (DEPRGs) between HCC samples and adjacent nontumorous samples (*P* < 0.05). Then, univariate Cox regression analysis was used to further identify prognosis-related DEPRGs. The expression of each gene (*i*) was adjusted to log2(*i* + 1) to increase the accuracy of the Cox regression results. In the univariate Cox regression analysis, FDR < 0.05 was set as the cut-off value. The Gene Ontology (GO) and Kyoto Encyclopedia of Genes and Genomes (KEGG) enrichment analyses were performed using the “http://org.Hs.eg.db” and “enrichplot” package.

### 2.3. Generation and Validation of the Signature

The LASSO Cox regression analysis was applied to identify hub DEPRGs to minimize the risk of overfitting among the signature [29, 30]. The independent variable in the regression was the normalized expression matrix of candidate prognostic DEPRGs, and the response variables were OS and the status of patients in TCGA cohort. Penalty parameter (*λ*) for the gene signature was determined by tenfold cross-validation following the minimum criteria (i.e., the value of *λ* corresponding to the lowest partial likelihood deviance). Thus, a total of five optimal genes were screened, and their relevant coefficients were calculated. Next, we used the following formula to calculate the risk score for each patient:

(1) $$\begin{array}{c} \text{risk score}=\text{expression for each gene}*\text{coefficient for each gene}. \end{array}$$

Based on the median risk score, all HCC patients were separated into high- and low-risk groups. The different OS between high- and low-risk groups was analyzed via the log rank test. The sensitivity and specificity of the signature were evaluated by time-dependent receiver operating characteristic (ROC) analysis.

The hazard proportional model was constructed by employing multivariate Cox regression analysis to determine the independent prognostic factors. A novel nomogram, including the risk score and other three clinical factors (age, gender, and TNM stage), was constructed to explore the proportional hazards assumption of the multivariate Cox model. Next, the calibration curves of the 1^st^, 2^nd^, and 3^rd^ years were generated to verify the accuracy of the nomogram. The correlation between risk scores and individual clinical characteristics was analyzed via Wilcoxon rank sum test.

### 2.4. Function Enrichment Analyses and Drug Sensitivity Test

All DEGs were screened between the high-risk and low-risk groups using the following filter (∣log2FC | ≥1, FDR < 0.05). The GO and KEGG enrichment analyses were performed based on the “clusterProfiler” R package. Estimated by the Gene Set Variation Analysis algorithm, the infiltrating score of 16 immune cells and the activity of 13 immune-related pathways were calculated. The half-maximal inhibitory concentration (IC_50_) was estimated by R package “pRRophetic” to evaluate the drug sensitivity [31]. The Connectivity Map (CMap) database (https://portals.broadinstitute.org/cmap/) was used to predict potential chemotherapeutic drugs.

### 2.5. Consensus Clustering Analyses

In the consensus clustering, the cumulative distribution function (CDF) and consistent matrix were used to evaluate the optimal number of subtypes [32]. Thus, two robust subtypes (clusters A and B) were obtained according to the transcription matrix of the five genes in the signature. The Kaplan-Meier survival curves were performed to analyze the OS of subtypes. The correlations between the subtypes, OS status, and risk score were explored using the “ggalluvial” R package.

### 2.6. Tumor Mutation Burden Correlation

The TMB score of each HCC patient in TCGA cohort was evaluated using the somatic mutation analysis. We constructed correlation scatter and boxplots based on the Pearson correlation analysis to search the effect of risk score on TMB. Waterfall plots regarding high- and low-risk groups were generated by R package “maftools.”

### 2.7. Immune Infiltration Analysis

The immune infiltration analysis was performed to calculate the correlation coefficient and construct bubble chart. The ESTIMATE algorithm was applied to derive the corresponding immune score, stromal score, and ESTIMATE score [33]. Next, the proportions of 22 immune cell types of HCC patients were calculated via the CIBERSORT algorithm.

### 2.8. Quantitative Real-Time PCR (qRT-PCR)

The total RNA from the liver tissues of the FAHWMU cohort was extracted using TRIzol reagent. The mRNA was then reverse transcribed into cDNA using ribo SCRIPTTM reverse transcription kit. The expression level of mRNA was calibrated with glyceraldehyde-3-phosphate dehydrogenase (GAPDH). The designed primers are shown in Table S2. SYBR Green master mix was added, and real-time PCR was carried out using a 7500 rapid quantitative PCR system (Applied Biosystems, USA). The CT value of each well was recorded, and the relative quantification of the amplified products was performed using the 2^−*Δ*Ct^ method.

### 2.9. Statistical Analysis

**Figure 1:**
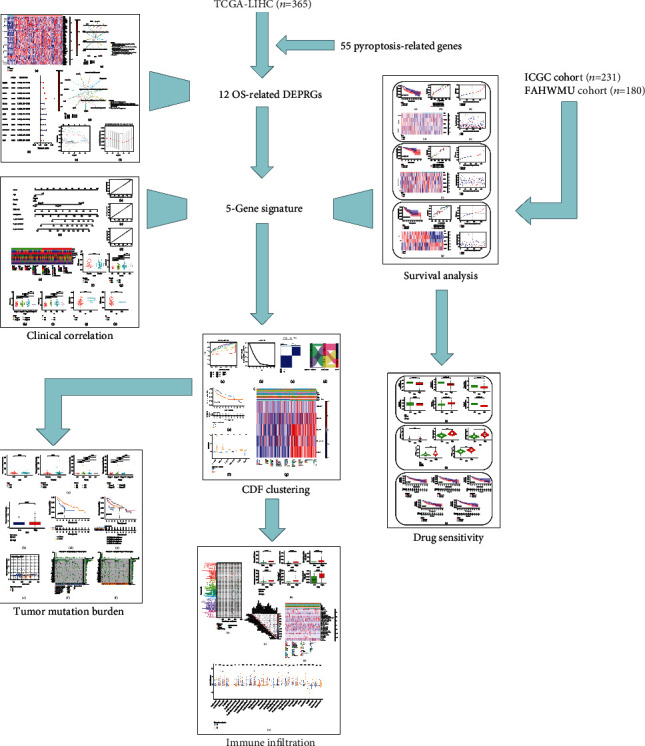
The flow chart of this study.

Here, the R version 3.6.1 (http://www.R-project.org) and its appropriate packages were used to perform all statistical analyses. *P* < 0.05 was considered as the standard of significantly statistical difference. The FDR method was used for multiple testing. Pearson test was used to compare the categorical variables. The overall workflow of this study is shown in Figure 1.

## 3. Results

### 3.1. Twelve Prognosis-Related DEPRGs Were Identified between Adjacent Nontumorous Samples and HCC Samples

**Figure 2:**
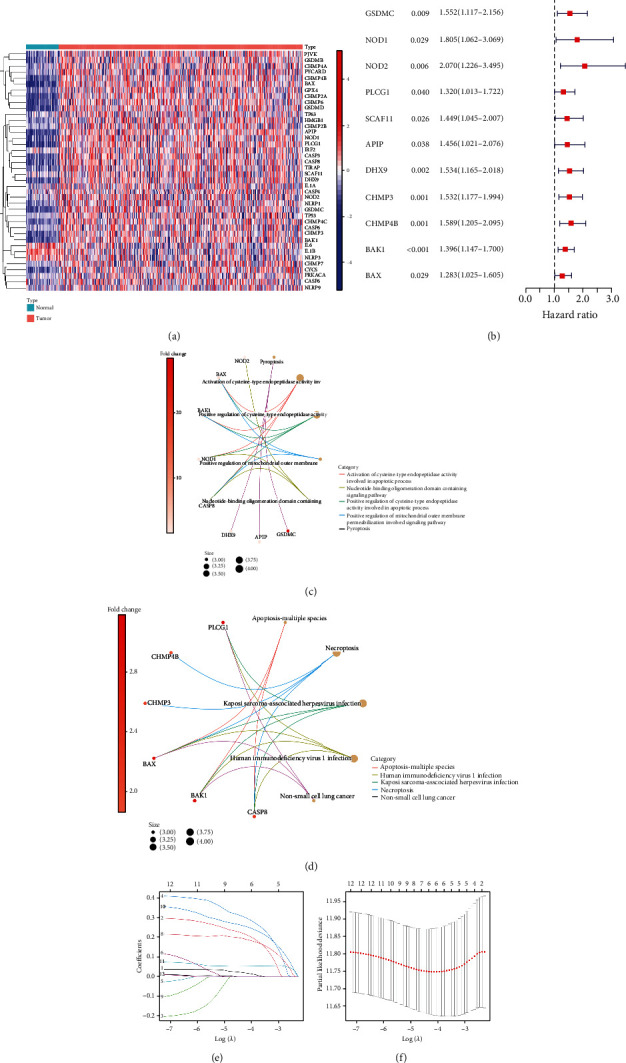
Identification of five optimal prognosis-related DEPRGs. (a) The heat map illustrated the differential expression of 40 PRGs between tumor samples and adjacent normal samples. (b) The Forest plot displayed the univariate Cox regression analyses regarding overall survival, and only DEPRGs with *P* < 0.05 and HR > 1 were regarded as prognosis-related DEPRGs. (c) CIRC plot for GO function enrichment of 12 prognosis-related DEPRGs. (d) CIRC plot for KEGG pathway enrichment of 12 prognosis-related DEPRGs. (e, f) LASSO regression analyses for screening five optimal PRGs.

The expressions of 55 PRGs were compared between 50 adjacent nontumorous and 365 HCC samples, and 40 DEGs were identified (*P* < 0.05). Among these, the expressions of 3 genes (IL6, IL1B, and NLRP3) were found to be downregulated in the tumor group while others were upregulated compared to the adjacent nontumorous group. The expression of these DEGs was shown in the heat maps (Figure 2(a)). The univariate Cox regression analyses further identified 12 DEPRGs regarding OS. The DEPRGs with *P* < 0.05 and hazard ratio (HR) > 1.000 were regarded as prognosis-related DEPRGs (Figure 2(b)). In addition, the GO enrichment analyses revealed that prognosis-related DEPRGs were mainly enriched in the pathways, including activation of cysteine−type endopeptidase activity involved in the apoptotic processes (Figure 2(c), *P* < 0.05 and *Q* < 0.05). The KEGG pathway enrichment plots demonstrated enrichment in Kaposi sarcoma-associated herpesvirus infection, necroptosis, and human immunodeficiency virus 1 infection (Figure 2(d), *P* < 0.05 and *Q* < 0.05). The overfitting of genes during the signature generation was prevented by LASSO regression analysis and finally identified the optimal five genes (GSDMC, DHX9, CHMP4B, BAK1, and NOD2) (Figures 2(e) and 2(f)).

### 3.2. Prognostic Value of the Signature Was Validated in TCGA Cohort and the Extra Validation Cohort

In TCGA cohort, using five optimal genes and the relevant coefficients, the risk score was calculated using the following formula:

(2) $$\begin{array}{c} \text{risk score}=\left(0.132*\text{GSDMC }\exp.\right)+\left(0.217*\text{NOD}2\;\exp.\right)+\left(0.149*\text{DHX}9\;\exp.\right)+\left(0.201*\text{CHMP}4\text{B }\exp.\right)+\left(0.055*\text{BAK}1\;\exp.\right) \end{array}$$

**Figure 3:**
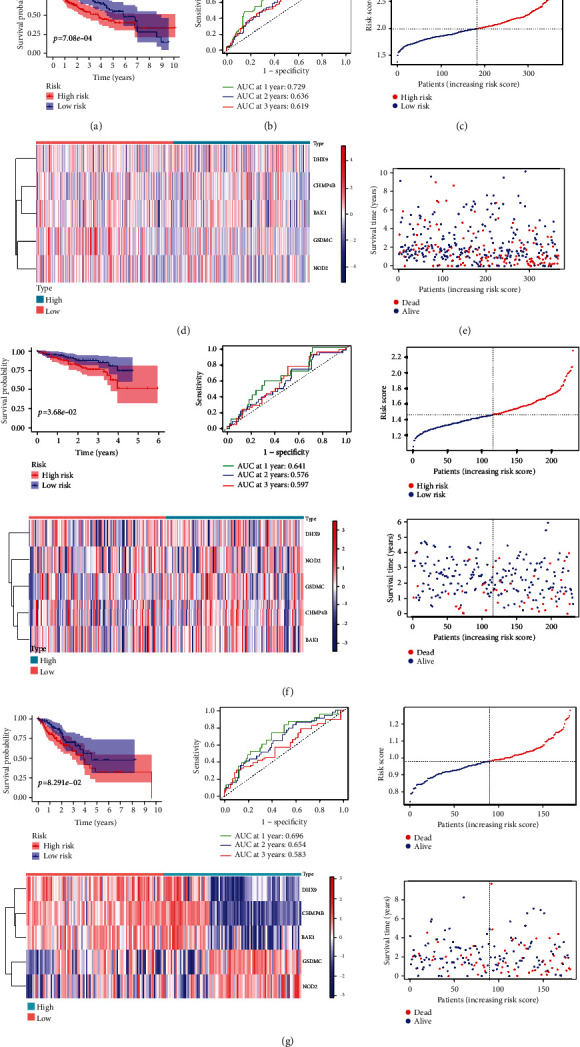
Generation and validation of the signature. (a) K-M survival curve displays the OS of patients between different risk groups in TCGA cohort. (b) Time-dependent ROC curves of the patients in TCGA cohort. (c) Heat map illustrated the expression patterns of five PRGs in TCGA cohort. (d) Distribution of the risk score in TCGA cohort. (e) Survival status scatter plots of patients in TCGA cohort. (f) Survival analysis with respect to the ICGC cohort (*n* = 231). (g) Survival analysis of the FAHWMU cohort (*n* = 180).

According to the median score calculated by the risk score formula, 365 patients were separated into low- and high-risk groups (Figures 3(c) and 3(d)). With an increase in the risk score, there was a gradual decrease in the survival time as well as an increase in the number of patients in death status (Figure 3(e)). Compared to the high-risk group, the low-risk group showed a better OS probability (Figure 3(a), *P* < 0.05). We found that the area under the ROC curve (AUC) reached 0.729 for 1^st^ year, and the AUC value for 2^nd^ and 3^rd^ years was both >0.600 (Figure 3(b)). HCC patients in the ICGC cohort and the FAHWMU cohort were also divided into high-risk group and low-risk group. The results of the survival analysis were similar to TCGA cohort (Figures 3(f) and 3(g)). Taken together, our results suggest the potential prognostic value of our signature in the prognosis of HCC patients.

### 3.3. Risk Score Was Identified as an Independent Prognostic Factor

**Figure 4:**
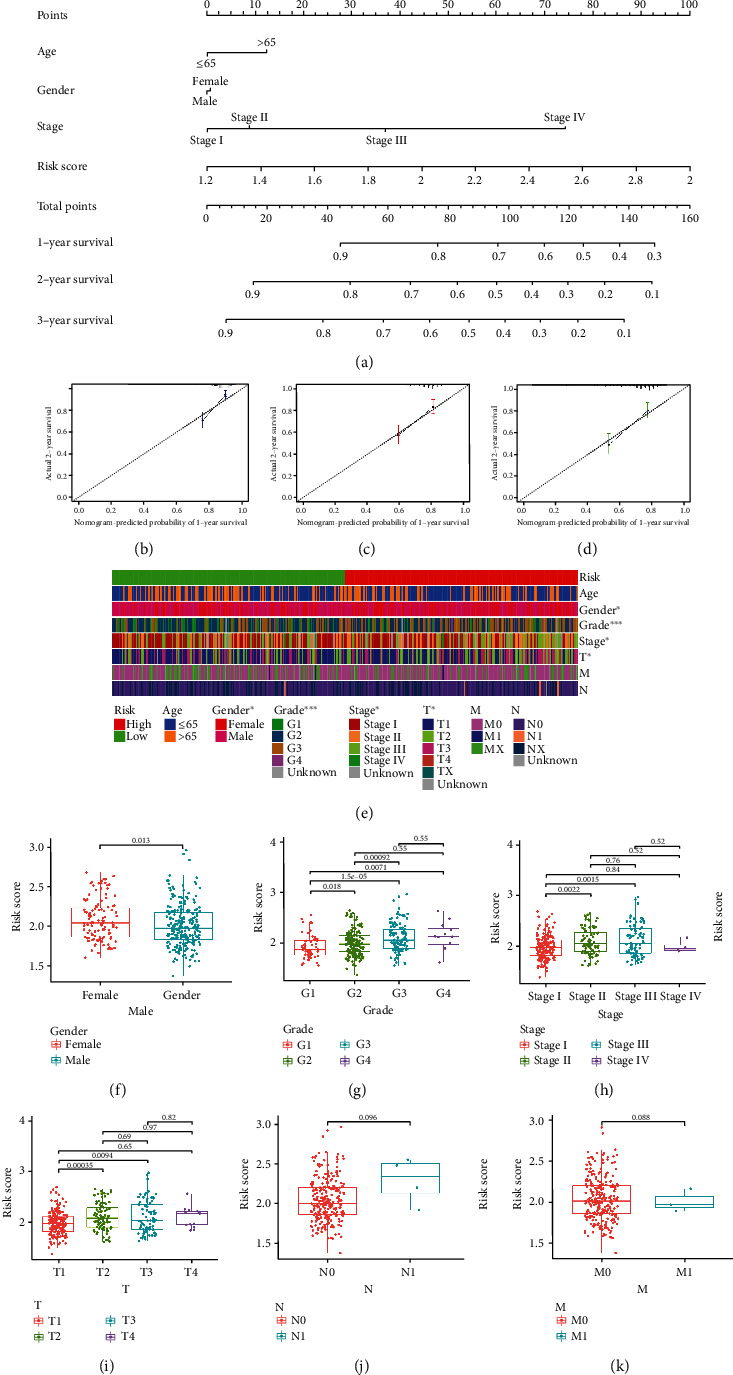
Selection of the independent prognostic factors. (a) The nomogram of the risk score and clinical parameters (age, gender, and TNM stage) in TCGA cohort. (b–d) The calibration curves displayed the accuracy of the nomogram in the 1^st^, 2^nd^, and 3^rd^ years. (e) Complex heat map illustrated the correlation between risk and clinical characteristics (^∗^*P* < 0.05, ^∗∗^*P* < 0.01, and ^∗∗∗^*P* < 0.001). (f–k) Boxplots demonstrated the differences in risk scores across clinical features (F: gender; G: grade; H: TNM stage; I: T stage; J: N stage; K: M stage).

In the univariate Cox analysis, the risk score and clinical factors (age, gender, and TNM stage) were significantly correlated with OS (Fig. S1A). Importantly, the risk score was confirmed as an independent predictor for OS in the multivariate Cox model (Fig. S1B). The OS-related nomogram was constructed to test the proportional hazards assumption in the multivariate Cox model (Figure 4(a)). Compared with other clinical factors, risk score had a better effect on the OS in the nomogram. With the integration of the risk score and clinical features (age, gender, and TNM stage), the 1^st^, 2^nd^, and 3^rd^ years of OS of patients with HCC could be predicted accurately. The subsequent calibration curves further verified the accuracy of the nomogram (Figures 4(b)–4(d)). The correlations between the risk and clinical characteristics (age, gender, tumor grade, TNM stage, T stage, N stage, and M stage) were shown in the complex heat map (Figure 4(e)). With increasing risk, the correlation between tumor grade and risk score was most significant (*P* < 0.001). The boxplot further confirmed that there was a significant upward trend of risk score with tumor grade from G1 to G4 (Figure 4(j), G1-G4: *P* = 0.0071). Additionally, with stage T from T1 to T3, the risk score was also significantly increased (Figure 4(i), T1-T3: *P* = 0.0094). The results of TNM stage were consistent with the results of stage I to stage III (Figure 4(h), stages I-III, *P* = 0.0015). Compared to the female patients with HCC, a lower risk score was found in male patients with HCC (Figure 4(f)).

### 3.4. Functional and the Immunological Activity Analyses

**Figure 5:**
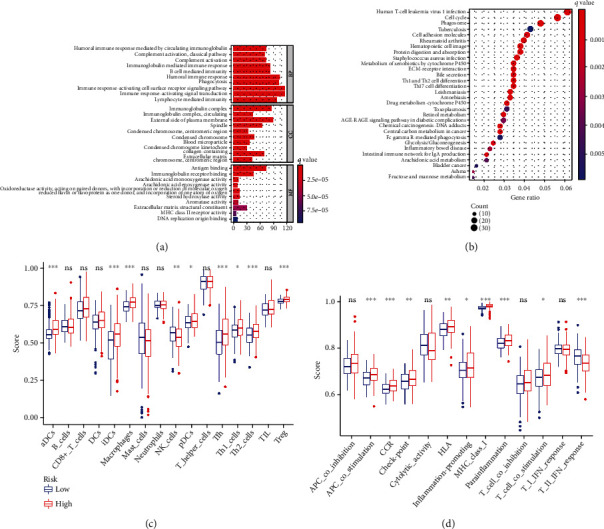
The enrichment analyses of the signature. (a) GO function enrichment of the signature (abscissa: number of DEGs, *P* < 0.05, *Q* < 0.05). (b) KEGG pathway enrichment of the signature (abscissa: number of DEGs, *P* < 0.05, *Q* < 0.05). (c) Comparison of the enrichment scores of 16 immune cells between different risk groups. (d) Comparison of the enrichment scores of 13 immune-related functions between different risk groups.

GO function enrichment and KEGG pathway enrichment were performed based on the risk score. In the results of GO enrichment, we found that the DEGs were mainly associated with immune response-activating cell surface receptor signaling pathway and immune response-activating signal transduction (Figure 5(a)), suggesting the involvement of immune infiltration in pyroptosis. KEGG pathway enrichment indicated that DEGs were mainly enriched in human T cell leukemia virus 1 infection, cell cycle, and phagosome (Figure 5(b)). ssGSEA was used to further analyze the scores of immune cells and immune-related pathways between the low- and high-risk groups. The scores of most immune cells (aDCs, DCs, iDCs, Tfh, Th2 cells, and Treg) were significantly increased with the increasing of risk (Figure 5(c), *P* < 0.001). In addition, the activity of 8 immune pathways in the high-risk group was higher than that in the low-risk group, except for the APC coinhibition, Cytolytic activity, T cell coinhibition, and type I IFN response (Figure 5(d)). Notably, type II IFN response was significantly lower in the high-risk group.

### 3.5. Drug Sensitivity Test Screened Six Potential Chemotherapy Drugs

**Figure 6:**
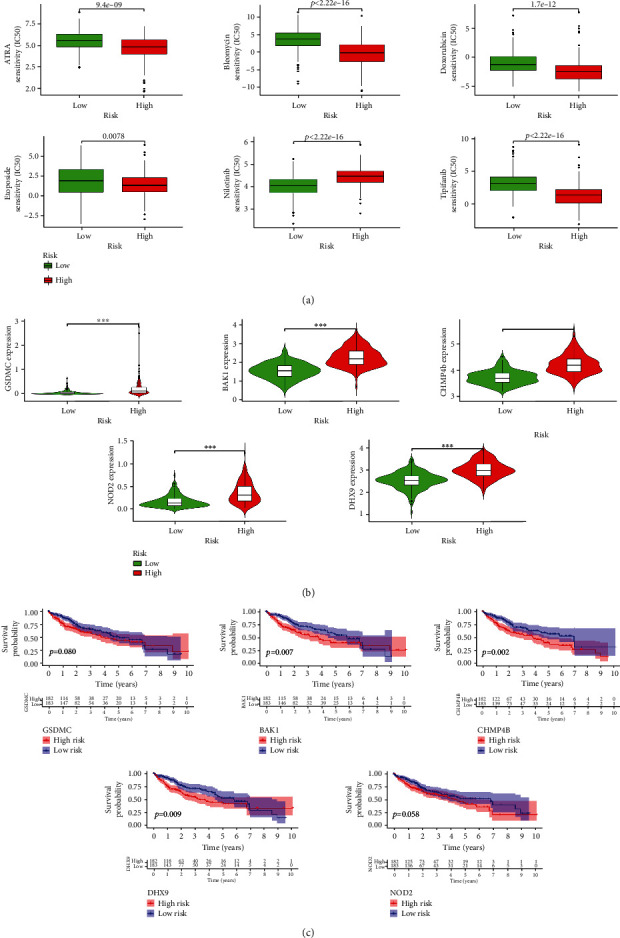
Drug sensitivity of the signature. (a) Boxplots displayed the estimated IC_50_ of different potential drug sensitivity (ATRA, Bleomycin, Doxorubicin, Etoposide, Nilotinib, and Tipifarnib) between low- and high-risk groups (all *P* < 0.05). (b) Violin plots presented significant differences in the expression of individual genes across the signature in high- and low-risk groups (^∗∗∗^*P* < 0.001). (c) K-M survival curves illustrated that the lower expression of individual genes across signature had better OS (*P* < 0.1).

The boxplots showed the results of drug sensitivity test (Figure 6(a)). By estimating IC_50_ between the low-risk and high-risk groups, 6 potential chemotherapy drugs were identified. The patients with HCC in the high-risk subtype showed obvious sensitivity to chemotherapy drugs, like ATRA, Bleomycin, Doxorubicin, Etoposide, Nilotinib, and Tipifarnib (all *P* < 0.05). Moreover, violin plots showed that individual genes in the signature were enhanced in the high-risk group (Figure 6(b)). Additionally, the K-M survival curves of individual genes in the signature showed a better OS in the low-expression group (Figure 6(c)).

### 3.6. Two Main Subtypes Were Divided Based on the Consensus Clustering Analysis

**Figure 7:**
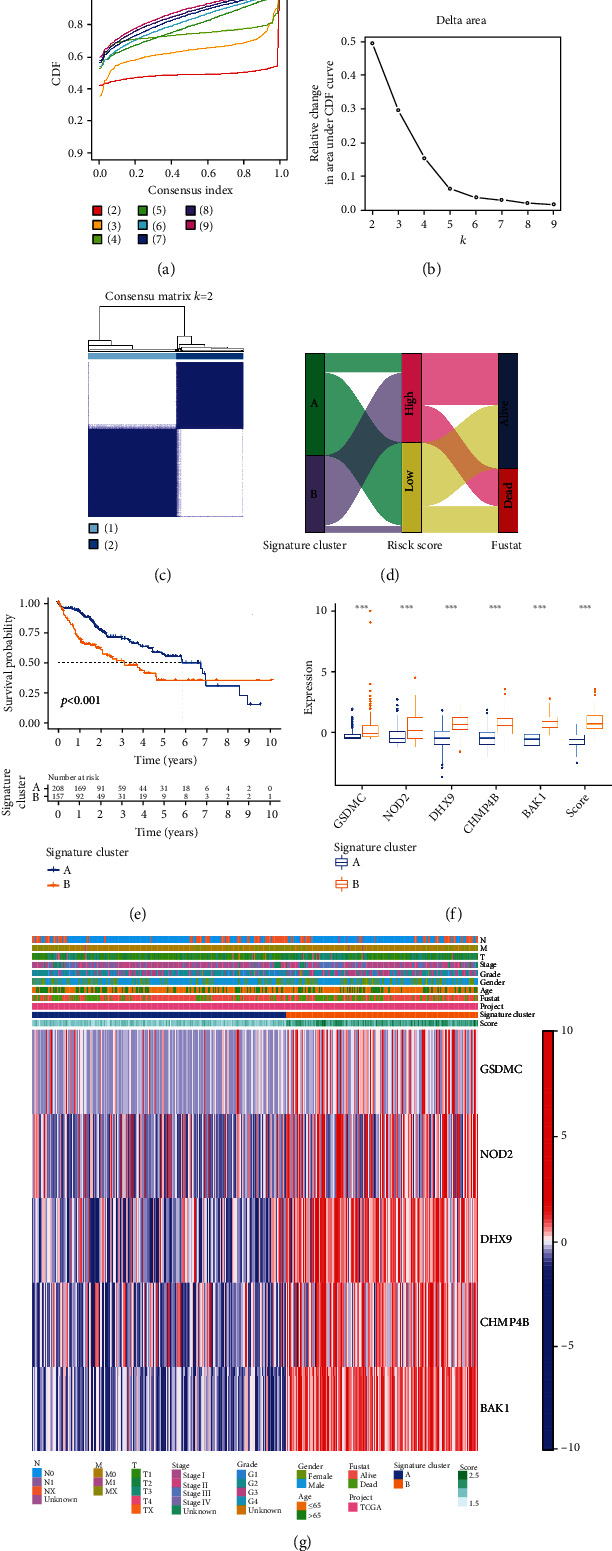
Clustering analyses of the signature. (a, b) Cumulative distribution function based on the signature indicated that the optimal number of subtypes was 2. (c) Concordance matrix of subtypes. (d) ggalluvial of two clusters displayed the correlation between clusters, risk, and survival status. (e) K-M survival curve of the two clusters. (f) Expression of individual genes across signature and risk score between cluster A and cluster B. (g) Complex heat map illustrated the expression patterns between cluster A and cluster B.

According to the *k* value selected by the highest cophenetic correlation coefficient, we divided all patients with HCC into two main subtypes (clusters A and B) (Figures 7(a)–7(c)). Compared to cluster A, patients in cluster B had higher risk scores and worse OS (Figures 7(d) and 7(e)). Moreover, all the expressions of individual genes were found to be higher in cluster B (Figure 7(f), *P* < 0.001). Combined with other clinical characteristics (age, gender, grade, TNM stage, T stage, N stage, and M stage) and risk score, the complex cluster-based heat map was constructed (Figure 7(g)).

### 3.7. The Potential Correlation between Signature and Tumor Mutation

**Figure 8:**
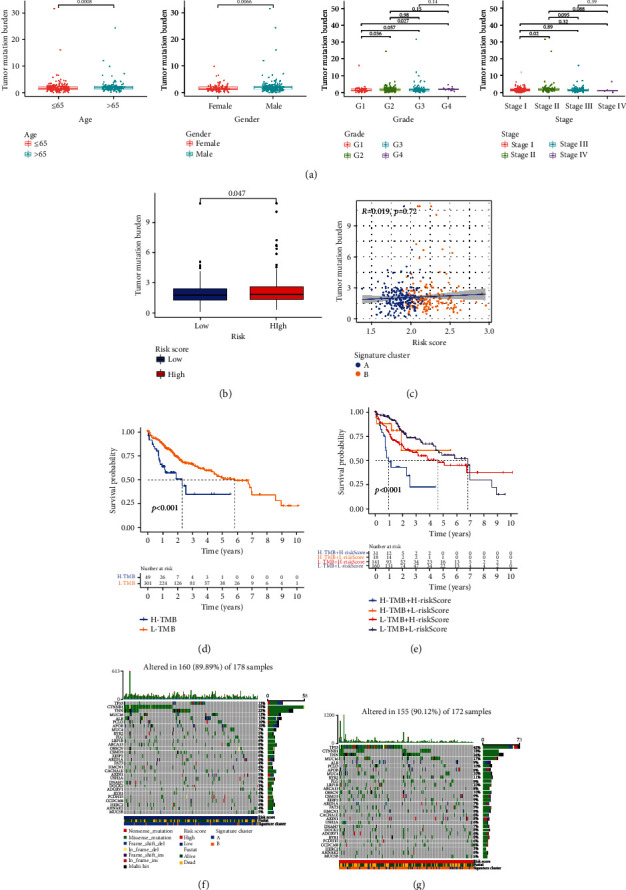
Tumor mutation burden analyses. (a) Boxplots showed the differences in TMB value among different clinical characteristics (age: *P* = 0.0008, gender: *P* = 0.0066, and G1-G4: *P* = 0.027). (b) Boxplot illustrated that the TMB value was significantly higher in the high-risk group (*P* = 0.047). (c) Scatter plot of correlations between the TMB value and the risk score. (d) K-M survival curve of the high-TMB group and the low-TMB group. (e) K-M survival curve of the comprehensive analyses between TMB and risk score. (f) Comparison of mutation frequencies in the low-risk group. (g) Comparison of mutation frequencies in the high-risk group.

The association between clinical features and TMB is shown in Figure 8(a). We found that patients with male and >65 y had a higher TMB value. TMB values were obviously increased with tumor grade from G1 to G4 and TNM stage from stages I to II. With an increase in risk score, TMB values were additionally increased (Figure 8(b), *P* = 0.047). Unfortunately, the correlation between TMB value and risk score was not significant (Figure 8(c), *R* = 0.019, *P* = 0.72). Our results showed that the higher level of TMB was observed in cluster A. Interestingly, K-M survival curves showed that the combination of higher TMB value and higher risk score was associated with worse OS (Figures 8(d) and 8(e), *P* < 0.001). Additionally, waterfall plots revealed that the mutation profiles of patients with HCC was lower in the low-risk group (Figures 8(f) and 8(g)).

### 3.8. Significant Correlation between Immune Infiltration and Signature

**Figure 9:**
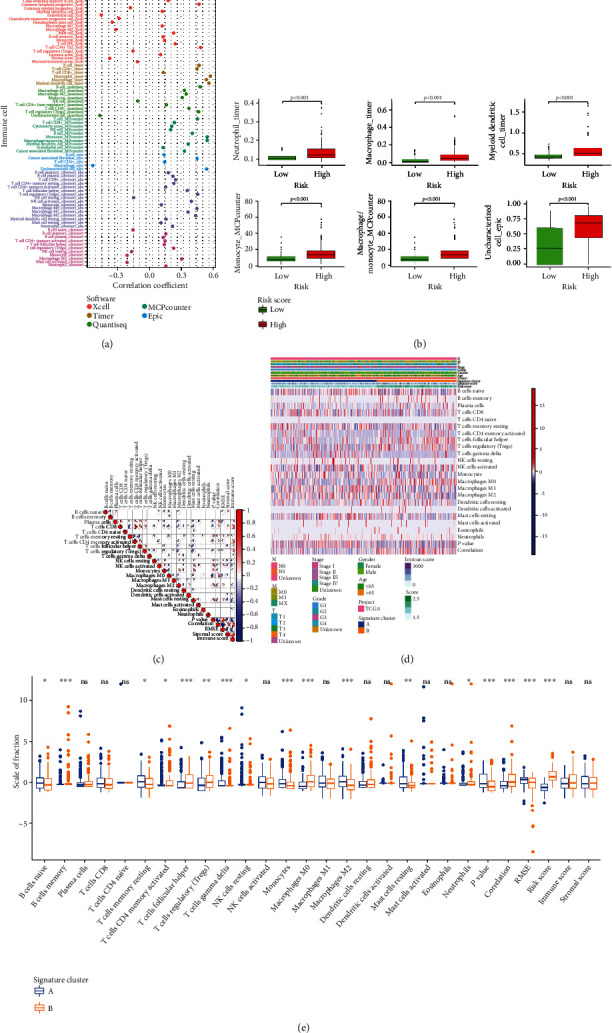
Immune correlation analyses and immune differential analyses of three clusters in the HCC patients. (a) Correlation coefficients between different immune cells and risk score. (b) Boxplots showed the content of immune cells with high correlation coefficients is significantly higher in the high-risk group. (c) The correlation plot of immune cells in TCGA cohort. (d) Complex heat map displayed the association between the expression levels of immune cells and clinical features in the HCC patients. (e) The differential analyses between immune cells and the scale of fraction for cluster A and cluster B.

Next, positive correlations were found between immune infiltration and risk score (Figure 9(a)). Boxplots showed that the contents of 6 immune cells were significantly higher in all the high-risk groups (Figure 9(b), all *P* < 0.001). Based on the ESTIMATE and CIBERSOFT algorithms, the proportions of 22 immune cell types in patients with HCC, the relevant ESTIMATE scores were calculated (Figure 9(c)). We found that the coefficients of immune cells including T cells and macrophages were significant. The complex heat map revealed the expression patterns of clinical features and the proportions of 22 immune cell types (Figure 9(d)). The boxplot showed the differences in scale of fraction of different immune cells in two clusters (Figure 9(e)).

## 4. Discussion

Recent studies have identified pyroptosis as a new form of programmed cell death, which plays an essential role in tumor development and treatment mechanisms [8]. Pyroptosis has been found to play a crucial role in various cancers, such as non-small-cell lung cancer and head and neck cancer [34, 35]. Currently, the pyroptosis-related prognostic signature has been constructed in ovarian cancer and gastric cancer, with an excellent prognostic potential [36, 37]. Thus, targeting PRGs may be a promising therapeutic strategy for HCC. However, comprehensive analysis of PRGs for prognosis prediction and targeted therapy in the patients with HCC still remains unclear. In the present study, we aimed to construct a novel prognostic risk signature and identify potential molecular subtypes to better predict the prognosis in HCC. The signature, which was validated by the ICGC cohort and the FAHWMU cohort, contributed to accurate prediction of the OS in patients with HCC. In addition, the high-risk patients identified by this signature were confirmed to be associated with higher TMB, drug sensitivity, and tumor immune cell content. Molecular subtypes (clusters A and B) were identified based on the signature. Further studies revealed that compared with cluster B, better survival outcomes and lower TMB were observed in cluster A. All these results suggest that this signature could serve as a new biomarker to improve the prognosis of HCC.

DHX9, CHMP4B, BAK1, NOD2, and GSDMC were the PRGs included in the prognostic signature. Wang et al. found that DHX9 could interact with CDK6 to promote the growth of HCC [38]. Elevated expression of CHMP4B has been found to play a key role in accelerating cell proliferation and resistance to doxorubicin in HCC [39]. In addition, elevated expression of BAK1 could exacerbate pyroptosis and further aggravate the invasion of HCC [40]. Hepatic NOD2, a well-characterized intracellular PRR of the NOD-like receptor (NLR) family, has been shown to promote hepatocarcinogenesis [41]. In addition, GSDMC, metabolized by *α*-ketoglutarate and mediated through caspase-8, results in pyroptosis [42]. Overall, increasing studies have confirmed the roles of these genes (DHX9, CHMP4B, BAK1, NOD2, and GSDMC) in HCC.

Previously, Hage et al. found that pyroptosis in macrophages mediates natural killer cell cytotoxicity against HCC [43]. In the present study, the effect of this signature on immune infiltration was also explored. Moreover, there was a positive correlation between macrophages under the TIMER database and risk score. The relative content of macrophages under the TIMER database was elevated in the high-risk group. It is known that drug therapy is crucial for the treatment of HCC [44, 45]. Previous studies have identified Doxorubicin as an effective drug to inhibit HCC via the regulation of apoptosis [46]. Herein, Doxorubicin was also confirmed as a potential drug against HCC, with higher drug sensitivity in the high-risk group. TMB, as a novel biomarker, has been intensively studied in precision medicine for HCC [47, 48]. Xu et al. found that TMB is positively correlated with clinical features in HCC [49]. Liu et al. found that LRP1B mutations are associated with higher TMB and poor prognosis in patients with HCC [50]. We also analyzed the correlation between TMB and the signature. Clearly, there was an obviously lower TMB value in the low-risk group, suggesting a potential correlation between TMB value and the signature.

There are several advantages in this study. First, the prognostic signature could accurately predict the OS for patients with HCC. In addition, the signature is significantly correlated with immune infiltration and TMB, suggesting its biomarker potential in HCC. The prognosis prediction of the signature is further confirmed by the FAHWMU cohort, suggesting its good prognostic prediction ability. More clinical samples are needed to validate the reliability of HCC prognostic value of this signature.

In conclusion, a novel prognostic PRG-signature is constructed for better prediction of prognosis in HCC, which may provide new insights into the treatment of HCC. In addition, this signature is closely associated with TMB and immune infiltration.

## Figures and Tables

**Table 1:** The clinical characteristics of TCGA cohort, ICGC cohort and FAHWMU cohort.

Variables	TCGA cohort (*N* = 365)	ICGC cohort (*N* = 231)	FAHWMU cohort (*N* = 180)
Survival status			
OS days (median)	556	780	803
Age (median)	61	69	64
Gender			
Female	119	61	114
Male	246	170	66
TNM stage			
Stage I	170	36	62
Stage II	84	105	50
Stage III	83	71	59
Stage IV	4	19	9
Unknown	24	0	0
Grade			
G1	55	NA	24
G2	175	NA	76
G3	118	NA	64
G4	12	NA	13
Unknown	5	NA	3

## Data Availability

The data and materials can be obtained by contacting the corresponding author.

## Abbreviations

HCC: Hepatocellular carcinoma
TCGA: The Cancer Genome Atlas
ICGC: International Cancer Genome Consortium
PRGs: Pyroptosis-related genes
LASSO: Least absolute shrinkage and selection operator
DEPRGs: Differentially expressed pyroptosis-related genes
PDEPRGs: Prognostic differentially expressed pyroptosis-related genes
FDR: False-discovery rate
OS: Overall survival
AUC: Area under the curve
ROC: Receiver operating characteristic
ssGSEA: Single-sample gene set enrichment analysis
K-M: Kaplan-Meier
GO: Gene Ontology
KEGG: Kyoto Encyclopedia of Genes and Genomes
CDF: Cumulative distribution function
TME: Tumor microenvironment
FAHWMU: The First Affiliated Hospital of Wenzhou Medical University.

## Supplementary Materials

Supplementary Materials Figure S1: univariate Cox analysis and multivariate Cox analysis containing risk score and clinical factors. Table S1: 55 pyroptosis-related genes used in this study. Table S2: list of primer sequences.
