# Supplementary material for: Signature Construction and Molecular Subtype Identification Based on Pyroptosis-Related Genes for Better Prediction of Prognosis in Hepatocellular Carcinoma
Source: Oxid Med Cell Longev. 2022 Jan 11;2022:4494713. doi: 10.1155/2022/4494713 (PMC8767411; doi:10.1155/2022/4494713)
Supplement: Supplementary Materials — Figure S1: univariate Cox analysis and multivariate Cox analysis containing risk score and clinical factors. Table S1: 55 pyroptosis-related genes used in this study. Table S2: list of primer sequences. [file 4494713.f1.zip › Supplemental Description.docx]

Supplemental Files:

**Table S1:** 55 pyroptosis-related genes used in this study

**Table S2.** List of primer sequences

**Fig. S1.** Univariate Cox analysis and multivariate Cox analysis containing risk score and clinical factors.
