# Supplementary material for: Signature Construction and Molecular Subtype Identification Based on Pyroptosis-Related Genes for Better Prediction of Prognosis in Hepatocellular Carcinoma
Source: Oxid Med Cell Longev. 2022 Jan 11;2022:4494713. doi: 10.1155/2022/4494713 (PMC8767411; doi:10.1155/2022/4494713)

Supplemental Files:

**Table S1:** 55 pyroptosis-related genes used in this study

AIM2

CASP1

CASP3

CASP4

CASP5

CASP6

CASP8

CASP9

ELANE

GPX4

GSDMA

GSDMB

GSDMC

GSDMD

IL18

IL1B

IL6

NLRC4

NLRP1

NLRP2

NLRP3

NLRP6

NLRP7

NOD1

NOD2

PJVK

PLCG1

PRKACA

PYCARD

SCAF11

TIRAP

TNF

APIP

DHX9

GZMA

NLRP9

ZBP1

NAIP

CHMP2A

CHMP2B

CHMP3

CHMP4A

CHMP4B

CHMP4C

CHMP6

CHMP7

CYCS

BAK1

BAX

IRF1

IRF2

TP53

TP63

HMGB1

IL1A

**Table S2.** List of primer sequences

| **Gene** | **Sequence (5'-3')** |  |
| --- | --- | --- |
|  |  |  |
| **DHX9** |  |  |
| Forward | CAGGAAGCGAAGGCTGATCT |  |
| Reverse | TTTGCCACACCAGGCATACA |  |
| **CHMP4B** |  |  |
| Forward | CCCGAAACAGTCCCTCTACC |  |
| Reverse | TCTTCTTTCTTCTTGGCGGGT |  |
| **BAK1** |  |  |
| Forward | GCACAGGGACAAGTAAAGGC |  |
| Reverse | ATGGGTCCCAGTGAAGTCTGC |  |
| **NOD2** |  |  |
| Forward | GTCCAGACCCTGCTCTTCAAC |  |
| Reverse | TCCTCAGGTACAGCTCGATG |  |
| **GSDMC** |  |  |
| Forward | GCAACAGGAGCTGGTAAGGA |  |
| Reverse | TGATGGCCAAACCCTCACTC |  |

**Fig. S1.** Univariate Cox analysis and multivariate Cox analysis containing risk score and clinical factors.


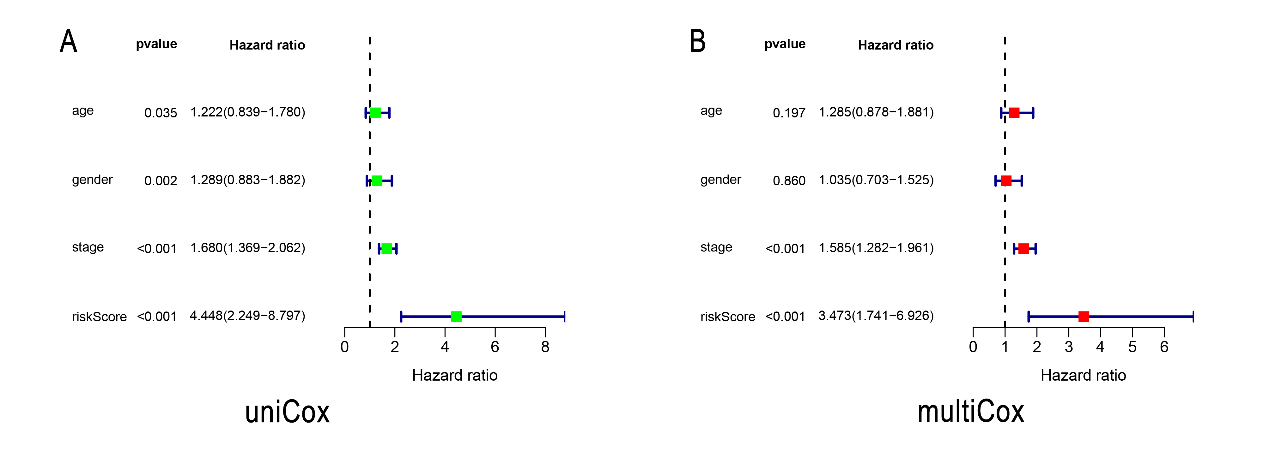

Supplement: Supplementary Materials — Figure S1: univariate Cox analysis and multivariate Cox analysis containing risk score and clinical factors. Table S1: 55 pyroptosis-related genes used in this study. Table S2: list of primer sequences. [file 4494713.f1.zip › Supplemental Files (2).docx]
